# Effect of maturation level on normative specific-agility performance metrics and their fitness predictors in soccer players aged 11–18 years

**DOI:** 10.1186/s13102-024-00855-z

**Published:** 2024-03-04

**Authors:** Mohamed Sellami, Emna Makni, Wassim Moalla, Raghad Tarwneh, Mohamed Elloumi

**Affiliations:** 1 Research Laboratory of Exercise Physiology and Pathophysiology (LR19ES09), Faculty of Medicine of Sousse. Sousse, University of Sousse, Sousse, Tunisia; 2 LR 19JS01 EM2S, Education, Motricity, Sport and Health, High Institute of Sport and Physical Education, University of Sfax, Sfax, Tunisia; 3 Sport Sciences and Diagnostics Research Group, GS-HPE Department, Prince Sultan University, Riyadh, Saudi Arabia

**Keywords:** Normative data, Change of direction, Young players, Soccer, Determinant factors

## Abstract

**Background:**

In sports sciences, normative data serve as standards for specific physical performance attributes, enhancing talent identification within a specific population. The aim of this study was to provide standard data for Agility-15 m, Ball-15 m tests, and skill index, considering maturation level, specifically age at peak height velocity (PHV). The study also investigated the relationship between relative performances in these tests and anthropometrics, jumping (squat jump [SJ], countermovement jump [CMJ]), and sprinting abilities (S-5 m, S-10 m, S-20 m, S-30 m) in young soccer players.

**Methods:**

The study involved 647 soccer players aged 11–18 years, categorized into three groups: pre-PHV, circum-PHV, and post-PHV. Statistical analysis was conducted using analysis of variance and Bonferroni post hoc testing to detect variations among maturation groups, and Pearson’s correlation test to examine the relationship between factors.

**Results:**

Agility-15 m and ball-15 m performances among maturity groups showed significant differences (*p* < 0.01). Significant correlations were identified in pre-PHV group, between lower limb length and Agility-15 m (*r*=-0.23, *p* < 0.05) and between height and Ball-15 m (*r*=-0.23, *p* < 0.05). In post-PHV group, there was a correlation between body fat percentage (BF %) and Agility-15 m (*r* = 0.17, *p* < 0.05) and Ball-15 m (*r* = 0.21, *p* < 0.05). In all maturity groups, there were significant correlations between agility and sprint tests (S-5 m, S-10 m, S-20 m, and S-30 m) and muscle power (SJ and CMJ).

**Conclusion:**

The study revealed that both speed and lower limb power significantly influence agility in young soccer players, providing valuable insights for coaches and practitioners to create tailored training plans and interventions for different age groups and maturity levels.

## Introduction

Soccer is the most popular team sport worldwide. During the game, soccer players engage in a variety of actions with intermittent high- and low-intensity intervals. These actions include short sprints, jumps, and directional changes, making player conditioning a complex process [1, 2]. In recent years, researchers, sports scientists, and practitioners have grown increasingly interested in understanding the key determinants of children’s and adolescents’ ability to change direction in youth soccer matches [3, 4]. Indeed, some authors define agility as a rapid whole-body movement that involves a change in speed or direction in response to an external stimulus (i.e., ball movement, opponent movement, and teammate movement) [4].

Considering the significance of agility as a factor of soccer performance [5], the soccer analysis primarily focused on the frequency of direction changes in each match. GPS analysis showed that soccer matches involve frequent direction changes. The most common activities in goal scenarios are sprinting and rapid acceleration [6], which happen every 2–4 s or 1200–1400 times [7]. Soccer fitness tests that assess a player’s ability to change direction are categorized as laboratory testing and soccer-specific field assessments. Despite the accuracy and effectiveness of the laboratory tests for assessing overall fitness levels, their relevance in evaluating soccer-specific skills has been challenged [8]. In this setting, agility performance in team sports, such as soccer, is assessed using several testing techniques [5, 9], including the agility t-test [10], modified agility t-test [11], Illinois change of direction test [12], modified Illinois change of direction test [13], and Agility-15 m test [14]. The latest agility test is an exclusive soccer-specific assessment that includes specific patterns such as forward and lateral movements and vertical jumps. The Agility-15 m test is an excellent method for assessing an athlete’s ability to change direction quickly and effectively, regardless of the ball possession [14]. It is also an effective method for calculating the Skill index, which is defined as decision-making when changing direction [15]. This index allows coaches and trainers to measure soccer performance and pinpoint areas that need improvement according to each age and maturity status. However, data on the development of agility and changes in direction in youth soccer players are limited. These data on player development, evaluation, and talent selection could be useful for trainers and coaches. It can also be used to evaluate players at different maturity stages and talent levels, enabling personalized training and performance improvement. However, several studies support dynamic talent identification and development training in sports, considering biological maturation level and peak height velocity (PHV) as standards [16, 17].

According to Loturco et al. [18], changes in direction speed were not affected in younger groups, but they decreased significantly in the adult soccer players Despite increasing neuromuscular training intensity and volume across different age groups. This suggests that a simple change to the strength-power training program during the maturation process may not be sufficient to improve adult players’ speed and agility [18]. Furthermore, Sheppard and Young [15] define agility assessment as a combination of physical and cognitive factors, including quickness to change direction and anticipation and pattern recognition. Assessing the agility of young soccer players can be a helpful reference point for enhancing performance and adjusting training programs based on their maturity and skill levels.

Accordingly, several studies have found an association between physical abilities such as strength, speed, and agility performance [11, 19]. Sariati et al. [20] recently showed an association between mental imagery and changes in direction in a small sample of young soccer players using the same agility test achieved in the current investigation. Furthermore, morphological changes in the athlete’s body during puberty have been proven to influence muscle mass and muscular strength, such as in vertical jumps and sprints [21]. Significant improvements in sprint and jump performance imply that puberty is a crucial period for developing physiological abilities [22]. In this regard, Radnor et al. [23] reported that post-PHV boys have significantly higher muscle architecture parameters than pre-PHV boys. However, agility performance can be influenced by physical parameters such as jumps and sprints, whether at starting or maximum speed. The current study will be tried to raise the question of relationship between maturity status and agility performance related. In this context, Haj Sassi et al. [11] identified an association between the modified agility t-test and free counter-movement jump and 10-m sprint performances for women, whereas, Kutlu et al. [24] showed a relationship between the classic t-test exercise and 20-m sprint performance. Although several studies have shown relationships between agility tests and physical testing, such as sprints and jumps [25–26–27], there is limited information in the literature about the pattern of performance and growth.

The study’s purpose was to establish normative metrics of a specific-agility test for soccer players aged 11–18 years at various phases of maturation and investigate the relationship between agility, vertical jumps, sprints, and anthropometric variables. We postulated that maturation’s influence on agility performance, mediated by positive effects on body dimensions and physical abilities, would vary across maturation stages.

## Materials and methods

### Participants

The a priori sample size was calculated using G*Power software (version 3.1.9.4; Kiel University, Kiel, Germany) [28]. We set α at 0.05 and power at 0.9. Based on subsequent discussions among the authors, we opted for a small effect size (*f* = 0.15). This allowed us to achieve the desired power with as few as 567 participants. However, to anticipate potential participant dropouts, we recruited 710 participants. Ultimately, 647 young soccer players (age 14 ± 1.91 years; body mass 56.72 ± 12.3 kg; height 166.5 ± 11.45 cm and body fat 13.41 ± 2.18% ) with a chronological age range of 11–18 years voluntarily took part in this investigation. The players belong to the same club in Tunisia’s top soccer league. They trained for ten months per year, engaging in an average of five training sessions and one match every week. To determine their maturity offset, the equation [29] applied in this study was based on predicting years from peak height velocity (PHV): Maturity offset = [(0.0036124 × age (years) × height (cm)] − 7.999994. Since the age at PHV is our reference, we describe each measurement in years concerning it defined as maturity offset [29].

A maturity offset of -1.0 indicates a player was measured 1 year before peak velocity, 0 during peak velocity, and + 1.0 after peak velocity [3, 30]. Three groups of players- pre-, circum-, and post-PHV- were formed. The study design was approved by the Faculty of Medicine of Sousse’s local research ethics committee on September 5, 2022, with approval number IRB00009014/2022. Written informed parental consent and participant consent were obtained in compliance with the Declaration of Helsinki. Participants underwent a comprehensive physical examination and received detailed information about the study’s methods, risks, and benefits.

### Procedures

#### Anthropometric measures

The anthropometric measurements were conducted by a single qualified anthropometrist with a PhD in physical anthropology and over 10 years of experience in this field. While not certified by the International Society for the Advancement of Kinanthropometry (ISAK), their qualifications and experience ensure adherence to established anthropometric measurement protocols. A digital balance (± 0.1 kg; ADE Electronic Column Scales, Hamburg, Germany) and a wall-mounted stadiometer (± 0.1 cm; Holtain Ltd., Crosswell, UK) were used to measure body mass and standing stature. According to a previously published methodology, every skinfold measurement was precisely positioned [31]. Every skinfold measurement was taken on the subject’s right side using a Harpenden skinfold caliper (+ 0.1 mm; Baty International, Burgess Hill, UK). Slaughter’s formula [32] was used to calculate body fat percentage (BF %) based on measurements of skinfold thicknesses (subscapular and triceps).

#### Agility and fitness tests

**Fig. 1:**
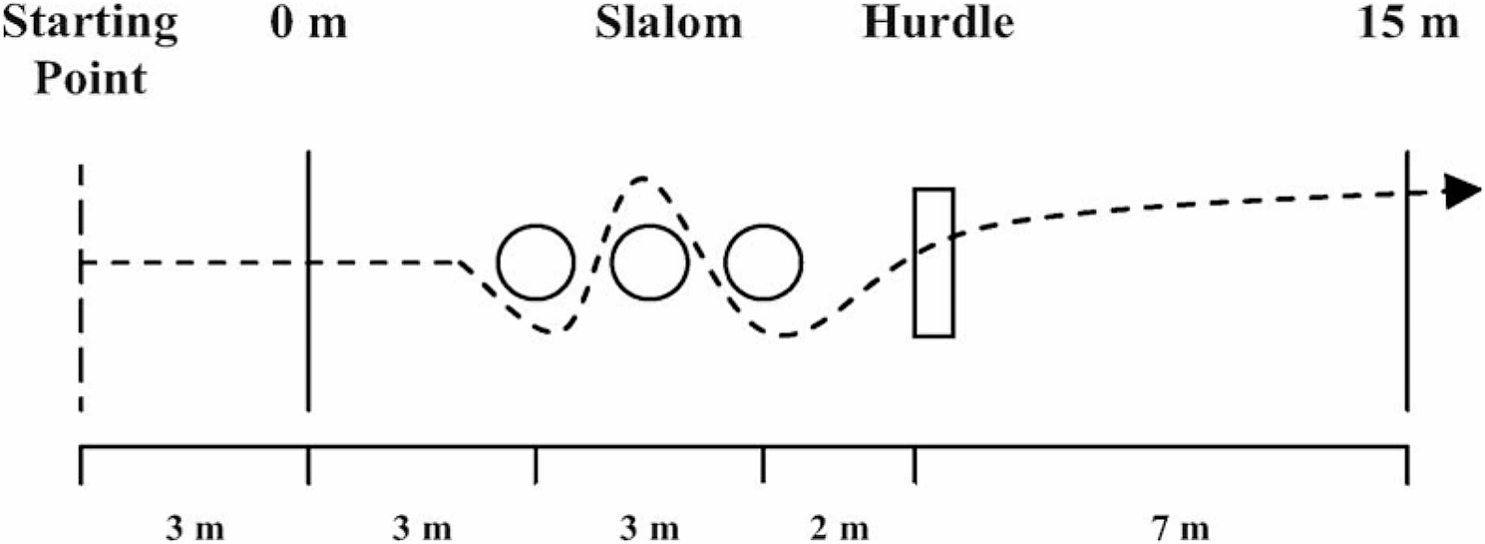
Schematic representation of the Agility-15 m test

The players performed the following agility and fitness tests: Agility-15 m and Ball-15 m tests (Fig. 1). For more details see Mujika et al. [14]. Each player performed two maximal Agility-15 m and Ball-15 m runs with a 3-min passive recovery period in between, and the best performance achieved during the run was selected for analysis. The skill index was determined as the time obtained from the Agility-15 m test with and without the ball. It was calculated as the ratio between Agility-15 m and Ball-15 m (Agility-15 m/Ball-15 m). A higher index was interpreted as a higher skill for controlling the ball, as recommended by Mirkov et al. [33].

In addition to agility tests, participants completed linear sprints over distances of 5, 10, 20, and 30 m and squat jump (SJ) and countermovement jump (CMJ) using photocells with barriers set placed on the starting line at a height of approximately 1 m and the Opto-jump system (MicrogateSARL, Italy), respectively. For more details, see Glatthorn et al. [34]. Players underwent a 15-minute warm-up consisting of low-intensity exercises, dynamic stretching, skipping, acceleration runs, and increasing-intensity jumping exercises before physical tests. Participants completed three trials with a 3-min passive recovery period in between, and the best performance was retained for analysis.

### Statistical analyses

The mean ± SD values are provided for anthropometric and physical fitness characteristics of the study participants. The study used the Statistical Package for Social Sciences (SPSS, version 20.0 for Windows; SPSS, Inc., Chicago, IL, USA) for statistical analyses with a significance level of *p* < 0.05. The Kolmogorov-Smirnov assessed the normality of the variables’ distribution. One-way analysis of variance explored differences between maturity groups in anthropometric variables and performance measurements. Group-specific differences were identified using Post hoc tests (Bonferroni procedure). Percentiles are used as normative data and centile curves from LMS Chart Maker Pro version 2.3 software (The Institute of Child Health, London, United Kingdom) [35]. The software normalizes the data using age-specific Box-Cox power transformations. Pearson’s product-moment correlation coefficients are used to assess relationships between variables. The strength of these correlations is categorized using Hopkins’ modified scale [36], with r ranging from trivial to perfect (0.1 trivial; 0.1–0.3 small; 0.3–0.5 moderate; 0.5–0.7 large; 0.7–0.9 very large; 0.9 nearly perfect; and 1 perfect).

## Results

Tables 1 and 2 display participants’ anthropometric characteristics and physical fitness performances and statistical variances across the different maturity groups (*p* < 0.001).

**Table 1:** Descriptive statistics (all in given in mean ± SD) of anthropometry and statistical differences from 3 groups of soccer players from different maturity groups

Maturity status				
	pre-PHV (*n* = 175)	circum-PHV (*n* = 225)	post-PHV (*n* = 247)	Total (*n* = 647)
Variables	(-3 to -1.0 y to PHV)	(-1 to 1.0 y to PHV)	(1 to 5 y to PHV)	(-3 to 5 y to PHV)
Experience (years)	7.97 ± 0.53	9.72 ± 0.73†	13.01 ± 1.37†	10.5 ± 2.31
Age (years)	11.47 ± 0.53	13.22 ± 0.73†	16.51 ± 1.37†	14 ± 1.91
Maturity offset (years)	-1.71 ± 0.42	0.08 ± 0.54†	2.58 ± 1.03†	0.49 ± 1.91
Height (cm)	151.86 ± 6.97	165.92 ± 5.78†	177.2 ± 5.38†	166.5 ± 11.45
Lower limb length (cm)	78.20 ± 2.90	83.53 ± 3.66†	89.93 ± 4.29†	84.5 ± 6.03
body mass (kg)	42.38 ± 6.66	54.48 ± 7.78†	69.6 ± 7.33†	56.72 ± 12.3
BMI (kg/m2)	18.36 ± 2.21	19.77 ± 2.39†	22.19 ± 2.22†	20.22 ± 2.75
Body Fat (%)	12.31 ± 1.1	14.21 ± 2.01†	13.42 ± 2.67†	13.41 ± 2.18
Lear mass (kg)	36.99 ± 5.48	46.72 ± 6.26†	60.19 ± 5.65†	49.19 ± 11.07

BMI: body mass index; †*p* < 0.001 between maturity groups

**Table 2:** Descriptive statistics (all in given in mean ± SD) of physical performance, and statistical differences from 3 groups of soccer players from different maturity groups

Maturity status				
	pre-PHV (*n* = 175)	circum-PHV (*n* = 225)	post-PHV (*n* = 247)	Total (*n* = 647)
Variables	(-3 to -1.0 y to PHV)	(-1 to 1.0 y to PHV)	(1 to 5 y to PHV)	(-3 to 5 y to PHV)
S-5 m (sec)	1.20 ± 0.13	1.16 ± 0.12†	1.13 ± 0.15†	1.16 ± 0.14
S-10 m (sec)	2.09 ± 0.15	1.98 ± 0.15†	1.86 ± 0.17†	1.96 ± 0.19
S-20 m (sec)	3.6 ± 0.20	3.37 ± 0.29†	3.10 ± 0.33†	3.33 ± 0.35
S-30 m (sec)	5.1 ± 0.24	4.80 ± 0.30†	4.41 ± 0.30†	4.73 ± 0.4
SJ (cm)	23.88 ± 4.08	26.66 ± 4.69†	32.88 ± 5.48†	28.28 ± 6.13
CMJ (cm)	25.74 ± 4.43	28.41 ± 4.75†	35 ± 5.85†	30.2 ± 6.42
Agility-15 m (sec)	4.06 ± 0.34	3.59 ± 0.30†	3.31 ± 0.25†	3.61 ± 0.42
Ball-15 m (sec)	5.24 ± 0.52	4.77 ± 0.50†	4.47 ± 0.38†	4.78 ± 0.56
Skill index	0.78 ± 0.05	0.76 ± 0.73†	0.74 ± 0.06	0.76 ± 0.06

PHV = peak height velocity; S-5 m = 5 m sprint; S-10 m = 10 m sprint; S-20 m = 20 m sprint; S-30 m = 30 m sprint; SJ = squat jump; CMJ = countermovement jump; Agility-15 m = Agility-15 m test; Ball-15 m = 15-m ball dribbling. †*p* < 0.001 between maturity groups

**Fig. 2:**
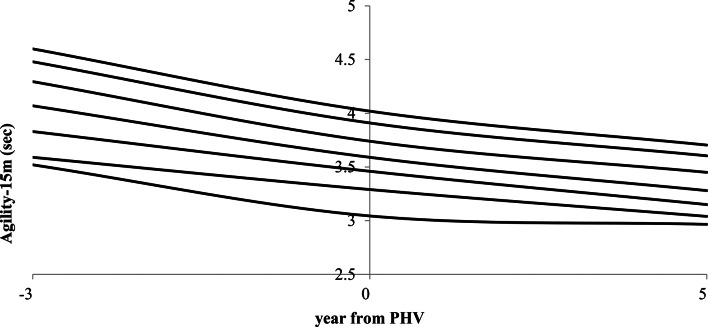
Smoothed LMS curves representing L (Lambda, skewness), M (Mu, median), and S (Sigma, coefficient of variation) for the 5th, 10th, 25th, 50th, 75th, 90th, and 95th percentiles of Agility-15 m in young soccer players from different biological age

Post-PHV players were older and taller, with more soccer experience, maturity offset, and body mass than pre-PHV and circum-PHV groups. In contrast, the pre-PHV group outperformed both post-PHV and circum-PHV groups in BMI (*p* < 0.001) and body fat (*p* < 0.001). Table 2 shows that post-PHV players performed best in fitness tests, while pre-PHV players performed worst. Significant differences were observed in Agility-15 m and Ball-15 m between maturity groups. The post-PHV group showed higher speed and agility than the pre-PHV group. Table 3 displays smoothed biological age category percentile values (5th, 10th, 25th, 50th, 75th, 90th, and 95th) for Agility-15 m and Ball-15 m performance, as well as Skill index values in young soccer players. Percentile values for Agility-15 m and Ball-15 m showed significant variations among maturity groups (*p* < 0.001). Figure 2 shows smoothed LMS curves for the 5th, 10th, 25th, 50th, 75th, 90th, and 95th percentiles of Agility-15 m in young soccer players of varied biological ages. Centile values were computed for each of the three maturation phases (pre-PHV, circum-PHV, and post-PHV).

**Table 3:** Smoothed biological age category percentile values for A-15 m and AB-15 m performances and Skill index in young soccer players aged 11–18 years

Maturity status				
	Percentile	pre-PHV (-3 to -1.0)	circum-PHV (-1 to 1.0)	post-PHV (1 to 5)
	5%	3.52	3.04	2.96
	10%	3.59	3.29	3.04
	25%	3.83	3.46	3.15
Agilty-15 m	50%	4.07	3.59	3.28
	75%	4.29	3.74	3.45
	90%	4.48	3.91	3.60
	95%	4.60	4.02	3.70
	5%	4.46	4.02	3.86
	10%	4.54	4.23	3.97
	25%	4.94	4.44	4.23
Ball-15 m	50%	5.23	4.71	4.48
	75%	5.58	5.05	4.47
	90%	5.91	5.47	4.91
	95%	6.12	5.71	5.03
	5%	0.70	0.66	0.59
	10%	0.77	0.80	0.79
	25%	0.93	0.94	1.00
Skill index	50%	1.15	1.14	1.14
	75%	1.37	1.36	1.33
	90%	1.65	1.72	1.57
	95%	1.84	1.87	1.69

Table 4 summarizes the correlations between anthropometrics, jumping, speed, and Agility-15 m and Ball-15 m performances in soccer players from different biological age categories. In post-PHV players height was significantly associated with Agility-15 m (*r*=-0.13, *p* < 0.05), while body fat was significantly correlated with both Agility-15 m and Ball-15 m in the post- (*r* = 0.17, *p* < 0.001; *r* = 0.21, *p* < 0.001). Likewise, lean mass was significantly correlated with Agility-15 m for the post-PHV (*r*=-0.13, *p* < 0.05), Ball-15 m for the circum-PHV group(*r*=-0.13; *p* < 0.05) and Skill Index for the post-PHV group (*r* = 0.16; *p* < 0.05). A significant correlation was observed between lower limb length and Agility-15 m for the pre-PHV group (*r*=-0.19; *p* < 0.05). However, no significant correlations were observed between body mass and BMI with Agility-15 m, Ball-15 m, and Skill Index for all maturity stages. Significant correlations were also observed between jumping (SJ and CMJ) and sprinting (5-m, 10-m, 20-m, and 30-m) performances and both Agility-15 m and Ball-15 m for all maturation stage groups. No significant correlations were observed between the 5-m sprint and the Skill index for the post-PHV group and all physical tests for the pre-PHV group; however, the 5-m sprint was correlated with the Skill index in the pre-PHV and circum-PHV groups. On the other hand, the Skill index was significantly correlated with the circum-PHV and post-PHV groups in the 10-m, 20-m, and 30-m sprints, SJ, and CMJ.

**Table 4:** Correlation coefficients between Agilty-15 m and Ball-15 m and skill index performance indices and anthropometry and physical performance from 3 groups of soccer players from different biological age categories (pre-PHV: -3 to -1.0 years to PHV; circum-PHV: -1.0 year to 1.0 year to PHV; and post-PHV: 1.0 to 5.0 years from PHV)

A-15 m				AB-15 m			Skill index			
	pre-PHV (-3 to -1.0)	circum-PHV (-1 to 1.0)	post-PHV (1 to 5)	pre-PHV (-3 to -1.0)	circum-PHV (-1 to 1.0)	post-PHV (1 to 5)	pre-PHV (-3 to -1.0)	circum-PHV (-1 to 1.0)	post-PHV (1 to 5)	
Body mass (kg)	-0.03	-0.1	-0.06	-0.04	-0.12	-0.12	-0.03	-0.12	-0.01	
Height (m)	-0.20	-0.006	-0.13*	-0.14	-0.11	-0.04	-0.03	-0.11	-0.03	
BMI (kg/m^2^)	0.12	-0.12	0.02	0.07	-0.08	0.01	-0.01	-0.12	-0.04	
Lean mass (kg)	-0.02	-0.12	-0.13*	-0.01	-0.13*	-0.09	0.002	-0.06	0.16*	
Body fat (%)	-0.05	0.009	0.17**	-0.04	0.01	0.21**	-0.01	-0.01	− 0.001	
Lower limb length (cm)	-0.19*	0.09	-0.08	-0.14	-0.02	-0.001	-0.03	-0.16*	-0.06	
S-5 m (sec)	0.53**	0.36**	0.54**	0.45**	0.33*	0.52**	0.18*	0.13*	-0.09	
S-10 m (sec)	0.45**	0.42**	0.43**	0.33**	0.41**	0.37**	0.06	0.32**	0.52**	
S-20 m (sec)	0.46**	0.33**	0.28**	0.35**	0.26**	0.14*	0.08	0.40**	0.37**	
S-30 m (sec)	0.46**	0.54**	0.37**	0.36**	0.45**	0.38**	0.1	0.26**	0.15*	
SJ (cm)	-0.44**	-0.41**	-0.5**	-0.28**	-0.35**	-0.52**	-0.01	0.45**	-0.37**	
CMJ (cm)	-0.40**	-0.42**	-0.48**	-0.29**	-0.35**	-0.5**	-0.06	-0.35**	-0.52**	

PHV = peak height velocity; A-15 m = Agility-15 m test; AB-15 m = 15-m ball dribbling; S-5 m = 5 m sprint; S-10 m = 10 m sprint; S-20 m = 20 m sprint; S-30 m = 30 m sprint; SJ = squat jump; CMJ = countermovement jump

*p < 0.05; **p < 0.01

## Discussion

Soccer demands a diverse range of agility skills, including rapid reaction times, precise technical movements, dynamic support, and the ability to quickly and effectively change direction, even under pressure from opponents or while controlling the ball [5]. This study aimed to collect standard data on a specific-agility test in a large sample of soccer players aged 11–18 years, considering their developmental phases and maturity status. The second aim was to analyze the relationship between anthropometric factors, agility (with and without ball), and sprinting and jumping abilities.

The main finding of this research presents percentile scores for Agility-15 m and reveals significant differences in this test results among young soccer players of varying maturity levels, with older players performing better. These results align with previous research by Negra et al. [27] and Peña-González et al. [21], who also observed a positive correlation between maturity level and agility performance, with players exhibiting advanced maturation achieving better results. One possible explanation for this is that players with advanced maturity are usually taller, heavier, and have superior physical performance in terms of jumping, sprinting, and changing direction abilities [21]. Maturation also affects agility performance through various physiological changes [22]. Particularly, agility performance in young soccer players can be affected by a variety of factors, including changes in the size of muscles, modifications to the fiber type composition, and modifications to fascicles and tendons [37]. The higher levels of testosterone and growth hormone associated with enhanced neural function, multi-joint coordination, and muscle power may also be responsible for the superior agility performance shown in players with advanced maturity [38].

This study also provides percentile values of the agility test in young soccer players, ranging from the 5th to 95th percentile, enabling coaches and physical trainers to assess their agility skills on a scale of 1 to 7. These provided reference values can serve as both standard data and baseline values for use in monitoring and identifying young soccer players’ capacity for accurately changing direction. The significance of speed and agility as differentiators in players of different performance levels has been shown by the consistent increase in speed displayed by elite soccer players over time [39]. The increasing emphasis on speed and agility has resulted in a number of research projects which attempt to find efficient exercises that improve soccer players’ agility. Likewise, significant correlations were observed between BF% and both Agility-15 m in post-PHV (*r* = 0.17, *p* < 0.01), and Ball-15 m in post-PHV (*r* = 0.21, *p* < 0.01). The findings are consistent with prior recent studies [3, 40], which have demonstrated a relationship between BF% and agility in their investigations. Similarly, Michael et al. [41] found a correlation between the T-test and fat percentage (*r* = 0.61. *p* < 0.01) in U14 soccer players. In the same context, data collected by the study by Stanković et al. [42] indicate that strikers who have a larger fat-free body mass ratio perform better on tests measuring their explosive strength and agility. However, there was a negative relationship between agility tests and BF% in elite female soccer players for midfield line players and defenders. As chronological age increases BF% decreases [40], allowing athletes to adjust their form and running technique in response to changes of direction. Excessive body fat in young athletes can impair muscular function and mobility, making it difficult to maintain high-intensity bursts of activity during soccer matches [21]. A higher BF% may lead to less opportunities for play, while a lower BF% may delay tiredness and allow players to maintain combined aerobic and anaerobic activity for extended periods [42]. To increase agility performance in young soccer players, coaches and fitness coaches should monitor the ideal body fat percentage in their players.

The study also found a significant positive correlation between Agility-15 m, Ball-15 m, and skill index with jumping and sprinting abilities across all mature groups (*r* = -0.28 to *r* = 0.54) (pre-PHV, circum-PHV, and post-PHV). This aligns with previous studies evaluating agility in soccer players. Negra et al. [27] reported moderate to high correlations between S-10 m performance and agility in young players, Chaleh et al. [25] found a correlation between agility and S-10 m performance in young players, and Papla et al. [43] found no correlation between agility and S-20 m sprinting in U16 soccer players. The discrepancy may be attributed to differences in the study design, participant characteristics, measurement techniques, and performance level that also could be considered as study limitations. The parallel evolution of speed and agility noted in the current study may be related to biomechanical and physiological factors, with faster sprint times indicating higher agility performance. It has been argued that players with greater sprint speed are more adaptable in agility tasks and able to execute sharper cuts and direction changes due to their ability to generate ground reaction forces [15]. Furthermore, from a physiological standpoint, both sprinting and agility movements rely on a combination of muscular strength, power, and coordination [15].

Another salient result is the significant relationship between participants’ agility and SJ and CMJ performances. These results corroborated previous investigations that found positive associations between anaerobic power and agility performance of young soccer players with varying maturity levels [25, 26]. In line with these findings, Köklü et al. [44] also observed a correlation between Zigzag agility without the ball and both SJ and CMJ performance. Lower body power and explosiveness, as assessed by these jumps, contribute to the athlete’s ability to generate quick and forceful movements. In this context, Hammami et al. [3] recommended that neuromuscular coordination, muscle strength, and power output could influence agility performance. Developing power and explosiveness in lower body movements, as demonstrated by SJ and CMJ, can enhance an athlete’s ability to change direction, accelerate, decelerate, and turn during a game [9]. However, coaches, fitness coaches, and practitioners should meticulously examine their training programs to enhance the physical fitness of young soccer players. Performing the same exercise or training volume may result in inadequate training impacts or injuries during the developmental stage [38].

### Study limitations

Previous studies have shown some discrepancy that may be attributed to differences in the study design, participant characteristics, measurement techniques, and performance level that could be considered as study limitations. Indeed, the experimental setting and methodological protocol including participants from the same club and region might be considered as the major limitation of the present study. Thus, including other participants from other regions and clubs could be interesting to confirm our results. Nevertheless, it is important to recognize that the data reported herein are representative of changes and responses of children related to the currently applied protocol. In the present study, soccer players maturity was related to performance and the lack of deep invasive measurements (i.e. biochemical dosage) and /or measurement of leg muscular power (i.e. isokinetic assessment) might be also considered as a second limitation. Therefore, caution is required when considering any results, particularly when comparing with different protocols, age and region characteristics.

### Practical applications

The study’s results can assist coaches and sports scientists in the assessment of the changes of direction and agility performance in young soccer players. Strength and condition fitness coaches should endorse physical training for young soccer players to address short-term disruptions or impairments in agility performance during their developmental period. In addition, when comparing different soccer groups, it is crucial to consider biological maturation or variations in muscle mass among players since difference are often shown due to growth. Thus, understanding maturity status and performance can provide coaches with valuable feedback on player progression and set a standard for evaluating agility at different maturity levels. This information could help trainers and strength conditioning coaches to provide more targeted guidance to their players.

## Conclusion

This study reveals that as young soccer players mature, they improve their agility performance, particularly in Agility-15 m and Ball-15 m tests. The study also found a significant relationship between agility and physical attributes such as height, lower limb length, body fat percentage, and sprinting and jumping abilities. These correlations highlight the importance of considering these factors in training programs to enhance agility in young players. Further investigations are warranted to understand the physiological and musculoskeletal changes underlying the variable relationships between anaerobic metabolism, explosive power, and agility in young team-sport athletes during maximal growth spurt, especially around peak of height velocity. Furthermore, it will also be interesting to investigate the physiological mechanisms underlying this relationship especially hormonal and metabolic adaptations.

## Data Availability

The data are available from the corresponding author upon reasonable request.
